# Public Speaking

**Published:** 1861-09

**Authors:** 


FROM THE MILLENIAL HARBINGER.

### PUBLIC SPEAKING.

*Health and Disease*—a Book for the People. By Dr. W. W. Hall, author of "Bronchitis and Kindred Diseases;" of "Consumption," and Editor of "Hall's Journal of Health." New York, A. D. 1859.

This is one of the most valuable books on *Health and Disease* that has fallen into my hands. It is written in a vigorous and transparent style, and is especially invaluable to public speakers, whether in-door or out-door speakers, and most especially invaluable to those called "preachers of the Gospel." It should be read and re-read by every preacher of the Gospel, and especially by the out-door speakers, of which class so many every year fall a prey to their ignorance and suicidal mannerisms in the violent inspirations and expirations of their lungs, or in one word, to their unnatural *respiration.* We have, at present, space only for the following extract:

"Public speakers, singers, auctioneers, etc., often bring on fatal diseases by the improper exercise of the vocal organs, and failing to protect them from cold immediately after. If a man speaks or sings in the air, or even in a house, where there is a current of air passing him, there are two causes of danger in operation. It requires more effort to speak in the open air, or in a draught, as in the hall, or passage, or stairway of a building; that effort debilitates the voice-organs sooner than he is aware, and with that effort and debility there is unnatural heat, while the current of air is constantly conveying the heat away from the body, depriving it of its natural amount, leaving the speaker or singer in the end weakened, exhausted, and if not really chilled, soon becomes so after ceasing the exercise. In all public speaking there is considerable muscular exertion, and always mental and bodily fatigue—sometimes almost exhaustion. The body perspires freely; it is not unfrequently that the inner garment is wet with perspiration. In this condition the body is chilled by very slight exposures; a very little wind, especially if the person stands still, or rides on horseback, or in a carriage, where there is no opportunity of muscular motion, is sufficient to bring on disease. To neglect the following precautions after exercising the vocal organs in a company, congregation, or other collection of persons, either in a parlor, public building, or in the open air, is suicidal. As soon as the exercises cease, put on an additional garment—shawl. coat cloak, or hat—and before leaving the building, especially in fire-time of year, bundle up well, put on gloves, close the mouth, pass out and walk on quickly. When the weather is decidedly cold, or damp, or windy, it is important to remain in the house five or ten minutes after the exercise, so as to allow the body to part with some of its heat, and the perspiration to subside or evaporate. The object of walking is to keep the blood in circulation and prevent a feeling of chilliness. The mouth should be kept closed, so that the cold air shall not pass directly to the throat and voice-organs, but shall be sent through the nose and head around to the throat and lungs, thus allowing it to get a little warmed in its circuitous route, before it reaches the delicate organs of voice. Valuable lives and good men would be saved every year by attention to these things. If a person feels the necessity of talking as he passes homeward, or if he finds he cannot walk fast enough to keep himself warm with the mouth closed, then hold a handkerchief in one hand, and place it over the nose and open mouth, not very closely, but so as to leave a little chamber for the mingling of the cold air from without with the warm air just passed. It may surprise any one to notice how much longer he may be kept warm in walking this way than if he talked freely without the above application. We knew a small, frail-looking clergyman, one who preached every night for weeks, if not months, together, and often in the day, in winter, in a densely crowded assembly, and yet, with the above precaution, never had even the slightest hoarseness. He was careful, however, as to another point; he always went to church and returned on foot and alone, so that there could be no temptation to neglect. The late Dr. Miller, that venerable and aged divine, Professor of Ecclesiastical History in the Theological Seminary of Princeton, New Jersey, while leaving his house to go to the seminary, in company with our brother, then a theological student, asked permission of him, on leaving his house, that he should be excused from "talking on the way," and at the same time placed a handkerchief before his face as above described, which he did not remove until he entered the threshold of the seminary. The former clergyman especially avoided going with ladies, having found it sometimes prevented him from walking fast enough to keep him sufficiently warm. These may seem to some trifling things, and an insufferable bother to attend to so many small matters; but nothing is trifling which saves human life, or averts years of sickness or suffering. The life of a single earnest worker in the ministry, fit for his place by education, piety, and a prudent mind, is worth more to the great world at large than the lives of a dozen senators, governors, or presidents. It is by the labors of such men that civilized governments stand. They 'are the salt of the earth; its preservative power.' As a President General once said to the writer, 'without religion this government cannot stand; we cannot do without churches.' And although a poor man, he subscribed five hundred dollars on the spot for the erection of a house of worship.

"Many valuable men are prematurely disabled, especially in the West and South-west, in consequence of having to ride a mile or two, or twenty, immediately after preaching; they think it to be a necessity. As long as the world stands, and the human constitution is under its present system of laws, this can never be done with impunity, and besides, the assertion that it is 'necessary,' is impertinent and untrue. Impertinent, because it implies that the Almighty cannot carry on the work of His kingdom without sacrificing His most efficient laborers. As to the 'necessity' of it, it cannot be necessary that a man should risk his life to be at any place an hour sooner or later. But more, there is a wicked presumption in such conduct, and a mawkish faith besides, in hoping that Providence will in some way or other preserve them from harm, inasmuch as they are about His work.

"The days of physical miracles have passed, and we may be very certain that the Almighty is not going to suspend a law of Nature to accommodate a preacher who is an hour behind his time. We cannot exactly see the 'necessity' for such a transaction. No law of Nature is a 'little thing,' nor is a violation of it 'little.' The theft of a pin, and the theft of a thousand dollars, are equally thefts. We many times do little things, subject ourselves to trifling exposures, with impunity, but that does not justify us in repeating such a thing deliberately. The reason that harm did not follow in every single case was simply because the effect was counteracted by some contingency. A man takes cold a dozen times, fifty or a hundred times, and it passes off without any striking consequences. The next time he takes a cold he expects it will pass off like the others; but sometimes it does not, it settles on the lungs, and he dies; yet he may have had worse colds before. Our highest wisdom and our only safety is in living up to the laws of our being all the time, habitually, and such are the persons who live to a good old age, in health of body, and in that cheerfulness of spirit which is a natural fruit of habitual health.

"For a zealous, warm-hearted, efficient minister of the Gospel to be 'laid on the shelf,' to be incompetent for ministerial labor in the very prime of life, amidst his usefulness, when the fields are already 'white to the harvest,' and the Macedonian cry, 'Come over and help us,' echoes from every quarter, like the wail of perishing mortals, is a 'burden hard to be borne,' even when incapacitated by causes wholly beyond his control. Charles Sumner, in writing from Aix, says: 'It is with a pang unspeakable that I find myself thus arrested in the labors of life, and in the duties of my position. This is harder to bear than the fire. I do not hear of friends engaged in active service without envy.' If a laborer for his country thus feels when incapacitated by violence from another's hands, how much more keenly must a laborer for the great God feel, when he must remain idle, while he sees others 'go in and possess the land.' Charles Sumner felt it to be a torture greater than of irons heated more than red hot, and applied to his naked flesh, with a view to his restoration, and that even in the service of an earthly master. But a torture how much more intense must it be to a single-hearted minister of the Gospel, and aggravated to a deeper depth, if his incapacity is the result of his own carelessness!

" *Voice-organs.* No experienced traveller or trainer of a race-horse starts his animal at full speed. He first walks, then trots, then gallops, as the animal thus holds out longer. All the muscles of all men and animals are under the same laws. It is by the movement of a variety of muscles about the throat that we speak. If, then, a person begins a song, or sermon, or harangue, on a high key, he will begin to cough and hem, and break down most rapidly; but if the same person begins in a low tone, in a conversational manner, and is at least eight or ten minutes in reaching the powers of his voice, he will speak much longer, and with scarcely appreciable fatigue. There is no need of commencing a sermon on a high key. A congregation instinctively adapts itself to the tone of the speaker, while their very effort at quietness favors their attention to the subject.

" A judge on the bench does not screech when he gives an opinion which consigns a fellow-man to a prison or a gibbet. The importance of the occasion produces an awe that stills every movement, silences every tongue, and makes the heart almost cease its beating. But greater interests than these are at stake when the minister of heaven stands up in the sacred desk, an ambassador of God as to things immortal. But he loses much of these advantages if he merely raves. Besides, in beginning boisterously he shoots off ahead of his hearers; they are not in sympathy with him, and the effect is in a measure lost. We should see daily much larger results from a preached Gospel if ministers could begin their services in a low tone, gradually increasing it, and then warm up more in unison with the people, it being always understood that the speaker has first thoroughly mastered his subject, as a distant object before his mind, his heart 'bound up' in the accomplishment of the object, with a feeling of deep and affectionate responsibility, in case that object is not accomplished from short-comings of his own.

"It is certainly a fact, that a man speaks with ease and effectiveness in proportion to his comprehension of his subject, and his interest in its promotion. It is said of one of the profoundest theologians and most effective preachers of a past age, that he spoke in a soft, slow, and low voice; the stillness made it awful, yet every hearer heard, while the waves of his eloquence swept across every heart. It is true he had a great mind to back him. We cannot say how mediocrity would fare in the premises.

"It is believed that an immeasurable amount of sickness, disease, and the weary suffering of weeks and months would be averted from multitudes every year, if the habitual precaution were taken, in weather even moderately cold, to close the mouth and breathe through the nose on going into a cooler atmosphere, and walking briskly for a few minutes, until the circulation has a little quickened, so as to get the blood started outwards, and gain momentum enough to resist the tendencies of the cold without, to drive it inward, and clog the machinery of life.

"This is a very little thing to remember, and an act very easily performed; while the omission of it, especially when coming out of a crowded apartment, warm to perspiration, and passing directly into a cold, raw, chilly atmosphere, brings death to many. But our recklessness of death does not allow us to hope that any considerable number of persons will take the trifling precaution which has been suggested."

We regard this extract worth more to every public speaker than the price of the volume. See pages 169 to 181. A. C.

---

WILLIAM CULLEN BRYANT, on his return from Europe, and after describing the small private residences of Paris, London, Boston, and Philadelphia, says, that "Fifth-avenue and Fourteenth-street (New York) are absolutely unsurpassed anywhere for the magnificence of their private dwellings."

